# Unveiling the secrets of marine—derived fucoidan for bone tissue engineering—A review

**DOI:** 10.3389/fbioe.2022.1100164

**Published:** 2023-01-09

**Authors:** Anupama Devi V. K., Anjaneyulu Udduttula, Amit Kumar Jaiswal

**Affiliations:** ^1^ Tissue Engineering Group, Centre for Biomaterials, Cellular and Molecular Theranostics (CBCMT), Vellore Institute of Technology (VIT), Vellore, Tamil Nadu, India; ^2^ School of Bio Sciences and Technology (SBST), Vellore Institute of Technology (VIT), Vellore, Tamil Nadu, India; ^3^ School of Engineering, Newcastle University, Newcastle Upon Tyne, United Kingdom

**Keywords:** biomaterials, polysaccharides, fucoidan, bone tissue engineering, bone regeneration

## Abstract

Biomedical uses for natural polysaccharides of marine origin are growing in popularity. The most prevalent polysaccharides, including alginates, agar, agarose and carrageenan, are found in seaweeds. One among these is fucoidan, which is a sulfated polysaccharide derived from brown algae. Compared to many of the biomaterials of marine origin currently in research, it is more broadly accessible and less expensive. This polysaccharide comes from the same family of brown algae from which alginate is extracted, but has garnered less research compared to it. Although it was the subject of research beginning in the 1910’s, not much has been done on it since then. Few researchers have focused on its potential for biomedical applications; nevertheless, a thorough knowledge of the molecular mechanisms behind its diverse features is still lacking. This review provides a quick outline of its history, sources, and organization. The characteristics of this potential biomaterial have also been explored, with a thorough analysis concentrating on its use in bone tissue engineering. With the preclinical research completed up to this point, the fucoidan research status globally has also been examined. Therefore, the study might be utilized as a comprehensive manual to understand in depth the research status of fucoidan, particularly for applications related to bone tissue engineering.

## 1 Introduction

One field that evolved in the 1990’s intending to restore missing or damaged tissues in the human body is tissue engineering (Sultan et al., 2021). It combined the interdisciplinary fields of research (cell sciences, biomaterials, and medical sciences), engineering, and mathematics. Making artificial organs and tissues, or “scaffolds,” that can replace the body’s injured parts is one of this field’s main objectives (Joshi et al., 2019).

**FIGURE 1:**
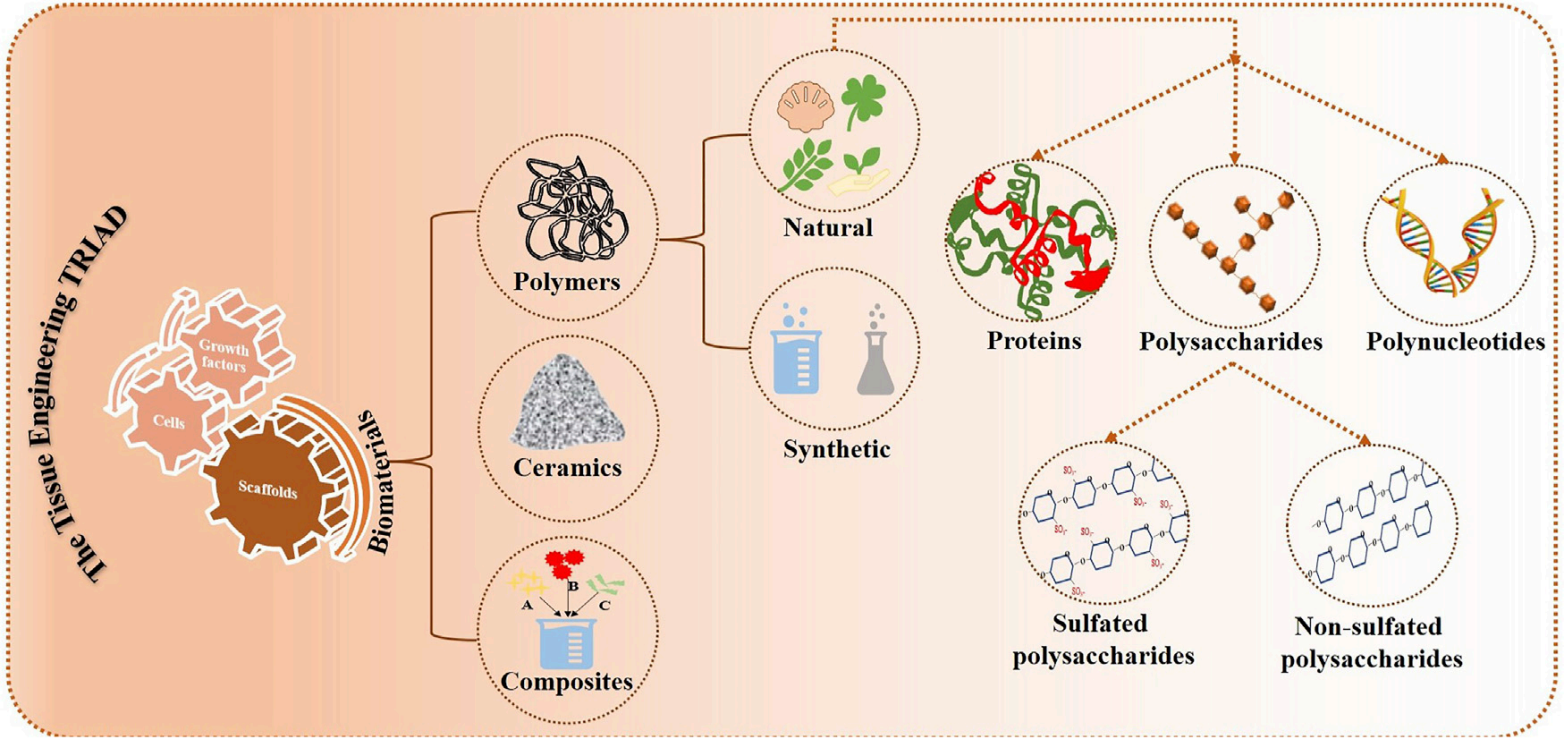
Various biomaterials in tissue engineering.

Fabricating a scaffold involves the use of biomaterials in a significant way. These biomaterials replicate the extracellular matrix of the cells to support surrounding tissues and organs and serve as the scaffold system or template that provides the necessary mechanical support for cellular interactions (O’Brien, 2011; R Choi, 2019). These biomaterials can be polymers, ceramics or composites. Among them are polymers, which are macromolecules formed by long chain backbones consisting of monomers and their side chains (the name “polymer” comes from the Greek word “polus,” which means many) (Namazi, 2017). The polymers are further divided into synthetic and natural polymers. Proteins, polynucleotides and polysaccharides are further divisions of the natural polymers (Paul et al., 2021). These classifications of biomaterials are depicted in Figure 1.

The simple sugar units known as polysaccharides, which are connected by glycosidic bonds, are structurally composed of homo- or hetero-monosaccharides and uronic acids (Maji, 2019; Ullah et al., 2019). Along with lipids, proteins, and polynucleotides, they are the fundamental macromolecules for all living things. They are also one of the frequently researched polymers that have long been of significant interest for biological purposes (Pattanashetti et al., 2021). The term “bioactive polysaccharides” refers to sugar-based substances that have undergone functionalization to create polysaccharides that have an impact on the biology of living organisms. Additionally, bioactive polysaccharides have been investigated as therapeutic agents against a variety of chronic diseases during the past few decades due to their biodegradability, non-toxicity, and biocompatibility. Studies on polysaccharides have shown a variety of pharmacological properties, such as antioxidative, anticancer, antibacterial, anti-obesity, hypolipidemic, antidiabetic, and hepatoprotective benefits (Ullah et al., 2019). These polysaccharides help a range of biological activities, including cell adhesion and differentiation, by interacting with other carbohydrates and proteins (Oldenkamp et al., 2019). Additionally, they aid in host defense, cell-cell identification, and intracellular fluid hydration (Venugopal, 2019). Since proteoglycans, glycosaminoglycan’s, glycoproteins, and glycolipids make up the majority of the extracellular matrix, polysaccharides have a crucial role to play in the selection of biomaterials for tissue engineering because of their biocompatibility, comparable structures and functions to many of these components, and their importance as biomolecular cues (Tchobanian et al., 2019).

These polysaccharides can be found in a variety of marine and terrestrial animals, including algae, seaweed, crustaceans, bacteria, and fungi. Due to their distinctive qualities, which include anticoagulant, immunological mediator, antioxidant, anti-tumor, anti-inflammatory, and antibacterial characteristics, they are extracted and explored for various applications (Joshi et al., 2019). Algal polysaccharides are a common type of polysaccharides, that can be further classified into sulfated polysaccharides and non-sulfated polysaccharides based on the presence or absence of a sulfate group in them (Venugopal, 2019).

**FIGURE 2:**
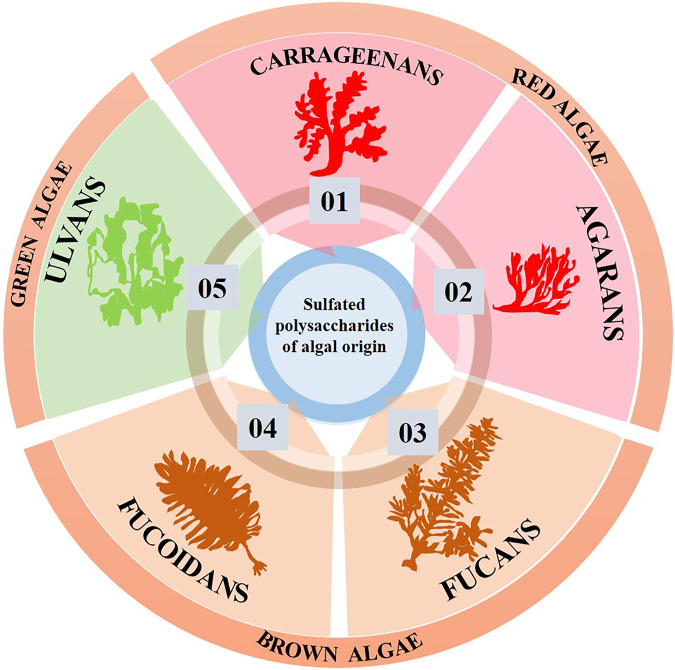
Sulfated polysaccharides of algal origin used in tissue engineering.

Sulfate groups are substituted for parts of the hydrogen bonds in the chain of the polysaccharide to form sulfated polysaccharides. The environment determines how these sulfate groups are replaced and arranged (Li et al., 2021). Polysaccharides of this type are more frequently found in marine species than those that originated on land. Chondroitin sulfate, fucoidan, keratin sulfate, carrageenan, and ulvans are a few examples of these sulfated polysaccharides (Li et al., 2021). Algae as such are classified based on color into Rhodophyceae (red algae), Phaeophyceae (brown algae), and Chlorophyceae (green algae) (Venugopal, 2019). Figure 2 shows various sulfated polysaccharides that are obtained from these marine algae. Excellent hydrocolloid properties allowed these polysaccharides to dissolve in water and turn into a viscous liquid. For instance, agar and carrageenan are great at forming hydrogels on their own, have excellent hydrocolloid characteristics, and can be used as stabilizers, emulsifiers. *etc*. (Muthukumar et al., 2021). One of these sulfated polysaccharides, fucoidan, has demonstrated some amazing features that point to its potential use in tissue engineering.

As a result, the main focus of this review is on understanding more about the polysaccharide known as fucoidan, along with a brief explanation of its origin, source, and structure. With a thorough analysis of the many areas in which it has been employed, particularly for the application of bone tissue engineering, the potential of this biomaterial has also been examined in terms of the properties displayed by it.

## 2 Fucoidan—An overview

### 2.1 Origin

Seaweeds are one of the main sources of polysaccharides, with roughly 70% of their dry weight made up of polysaccharides (Venugopal, 2019). Polysaccharides made from seaweeds include amylose, alginate, agar, agarose, carrageenan, and chitosan (Venkatesan et al., 2019). These are made from a variety of seaweed species, including algae, crabs, and others. Among these, the sulfated polysaccharide fucoidan is found in brown seaweed (Zayed and Ulber, 2019; Kuznetsova et al., 2020).

In 1913, Kylin discovered this polysaccharide for the first time and gave it the name “fucoidin.” Later, with the implementation of IUPAC regulations, it was given the name “fucoidan,” also known as “sulfated fucan,” “fucan,” and “fucosan” (Li et al., 2008; Wang et al., 2019; Zayed and Ulber, 2019). Fucoidan’s numerous medicinal and pharmacological applications have now been extensively researched. Numerous biological qualities, such as anti-tumor, antithrombotic, antiviral, immunomodulatory, anti-inflammatory, and several other properties were reported to be present in it (Li et al., 2008). In addition to being used for medical purposes, it is also commonly consumed in Japan, China, and South Korea (Wang et al., 2019).

### 2.2 Sources and types

**FIGURE 3:**
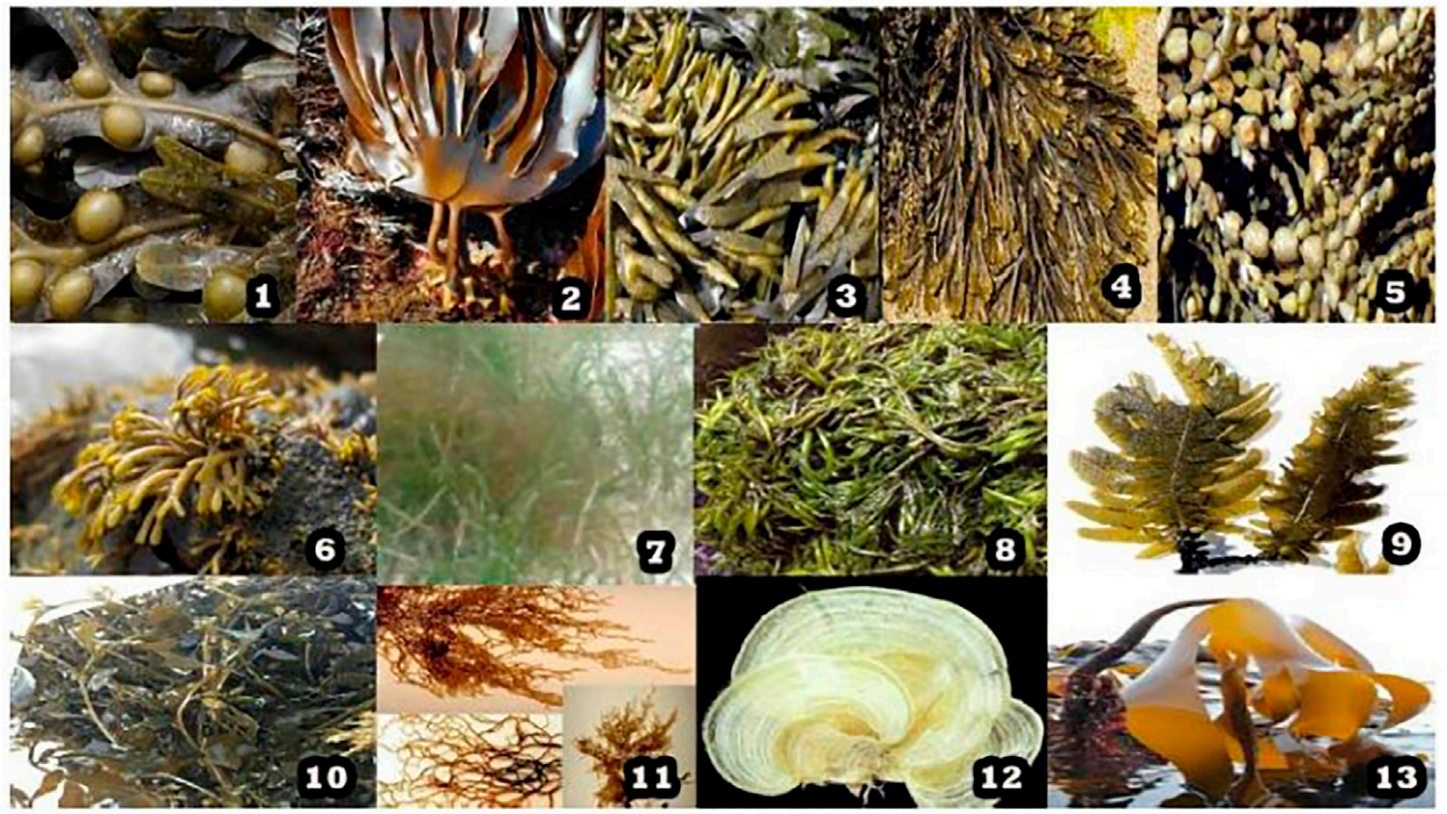
Various marine sources of sulfated polysaccharide - fucoidan, where 1. *Fucus vesiculosus*, 2. *Laminaria digitata*, 3. *Fucus evanescens*, 4. *Fucus serratus*, 5. *Ascophyllum nodosum*, 6. *Pelvetia canaliculata*, 7. *Cladosiphon okamuranus*, 8. *Sargassum fusiforme*, 9. *Laminaria japonica*, 10. *Sargassum horneri*, 11. *Nemacystus decipiens*, 12. *Padina gymnospora*, 13. *Laminaria hyperborea*. [Reproduced with permission from Ref (Luthuli et al., 2019) under Creative Commons Attribution License (CC BY)].

Fucoidans have been explored more and more recently to develop drugs or functional meals because they are more accessible than other sulfated polysaccharides from a range of affordable sources (Li et al., 2008). These are derived from the cell walls of brown seaweed and marine animals (Wang et al., 2019). Various sources of fucoidan have been shown in Figure 3. *Ascophyllum nodosum, Pelvetia canaliculata, Cladosiphon okamuranus, Sargassum fusiforme, Laminaria japonica, Sargassum horneri, Nemacystus decipiens, Padina gymnospora, and Laminaria hyperborea* are only a few examples of the many species from which these polysaccharides could be obtained (Luthuli et al., 2019).

Seaweed is composed of microscopic, multicellular marine algae. Due to their composition in proteins, vitamins, carotenoids, essential fatty acids, minerals, dietary fibres, and several functional bioactive components like polyphenols, polysaccharides, and peptides, seaweeds provide distinct nutritional and physiological benefits for livestock and fish species (Abdel-Latif et al., 2022). Fucoidan is found in algal cell walls and is also frequently isolated from other brown seaweeds (Joshi et al., 2019). It’s concentrations vary from species to species, for example, 19.0% in *Ecklonia radiata* to 51.2% in *Cladosiphon* sp (Abdel-Latif et al., 2022).

As discussed earlier sulfated polysaccharides are abundant in algae. The polysaccharides used to make biomaterials, including agar, carrageenan, xylans, floridean starch (an amylopectin-like glucan), water-soluble sulfated galactan, and porphyrin, are found in the red algae family Rhodophyceae. Alginic acid, laminarin, and the sulfated heteropolysaccharide sargassum are found in the brown algal family Phaeophyceae, whereas sulfated ulvans and xylans are found in the green algae family Chlorophyceae (Venugopal, 2019; Abdel-Latif et al., 2022).

In addition, there are more sources listed in the literature. The northern hemisphere kelp species *Fucus vesiculosis* is the source of experimental fucoidan, which is utilized most frequently in the literature. Due to its favorable toxicity profile and convenient availability throughout Asia, this has been frequently employed. Here, the skeleton sugars have shifted. For instance, *Undaria pinnatifida* fucoidan has a significant amount of galactose (Fitton, 2011; Fitton et al., 2015).

### 2.3 Structure of fucoidan

**FIGURE 4:**
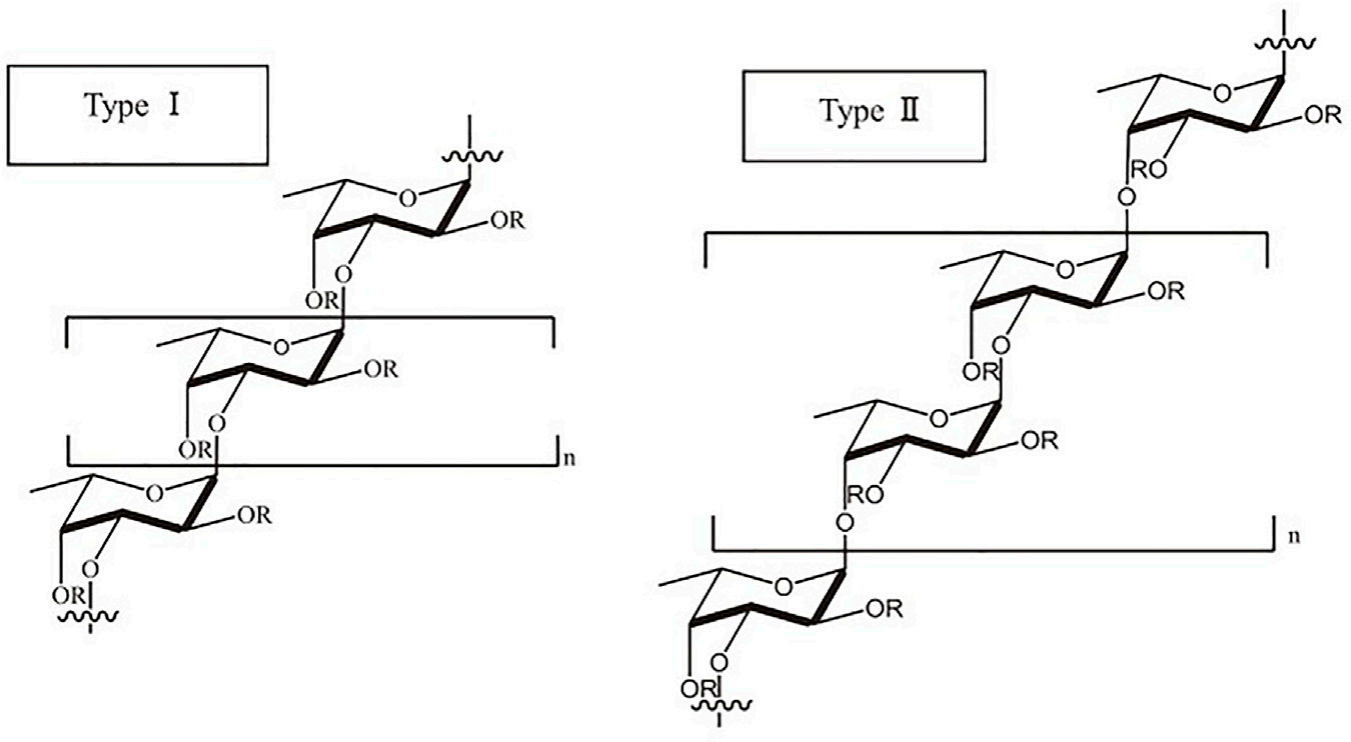
Type I and type II chains of fucoidan. [Reproduced with permission from Ref (Luthuli et al., 2019) under Creative Commons Attribution License (CC BY)].

Fucoidan, a water-soluble heteropolysaccharide with L-fucose-4-sulfate as its primary monosaccharide, is predominantly composed of extra L-fucose and sulphate groups. Consequently, deoxysucrose fucose is acknowledged as the primary constituent of the polysaccharide. It also contains galactose, xylose, mannose, rhamnose, glucose, arabinose, and uronic acid among other monosaccharides (Abdel-Latif et al., 2022). As a result, it contains two distinct chain structures: one that contains L fucopyranose and the other monosaccharide unit. These are composed of 1-3 connected sulfated L-fucose with an alternating pattern of 1-3 and 1-4 glycosidic linkages that repeats (Joshi et al., 2019; Apostolova et al., 2020). Branches have been replaced in some fucoidans at sites C-2 and C-3. The 1,3-linked L-fucopyranose residues are repeated in type I chains, while the 1, 4-linked L-fucopyranose residues are alternated in type II chains. The type I and type II chain structures of fucoidan has been shown in Figure 4. Additionally, the monosaccharides of fucoidan are joined by glycosidic linkages at positions 1, 2, 3, or 4, and sulphation may also occur at positions 2, 3, and 4 (Abdel-Latif et al., 2022). The fucoidan structure may also contain a range of monosaccharides (mannose, galactose, arabinose, xylose, glucose, *etc.*), uronic acids, and proteins in addition to the fucosyl main chain (Yunhai et al., 2018; Apostolova et al., 2020).

Depending on the source and the extraction technique used, it has a molecular weight range of 10,000 to 100,000 Da and an average molecular weight of 20 kDa. The type of seaweed selected, the source’s location, the anatomical region, and the season in which it is collected are often related to the composition and structure, just as the molecular weight varies (Joshi et al., 2019; Wang et al., 2019; Abdel-Latif et al., 2022).

## 3 Properties of fucoidan

Fucoidan has been found to have biological qualities that are appealing to people, including anticancer, antiviral, antiallergenic, anticoagulant, antioxidant, anti-inflammatory, immuno-modulatory, cardioprotective, and hepatoprotective effects (Zayed and Ulber, 2019; Li et al., 2021; Abdel-Latif et al., 2022). It is a constituent in many biological processes, including cell migration, adhesion, proliferation, and the occurrence of anticoagulant and antithrombotic characteristics. Additionally, it binds to and regulates the activity of proangiogenic growth factors like fibroblast growth factor (FGF), enhances endothelial cell qualities, inhibits the release of plasminogen activator inhibitor-1, and regulates cell surface when growth factor is present (Joshi et al., 2019). The structure of fucoidan resembles the human extracellular matrix, further due to its high biocompatibility, biodegradability, low toxicity, renewability, moisture-retaining ability, swelling ability, and colloidal properties, it has gained the attention of the research community in recent times. It can be employed to develop a range of regenerative medicine materials, such as those that are employed in the treatment of wounds. Based on polysaccharides, numerous wound dressings with a variety of applications have also been developed (Kuznetsova et al., 2020).

**FIGURE 5:**
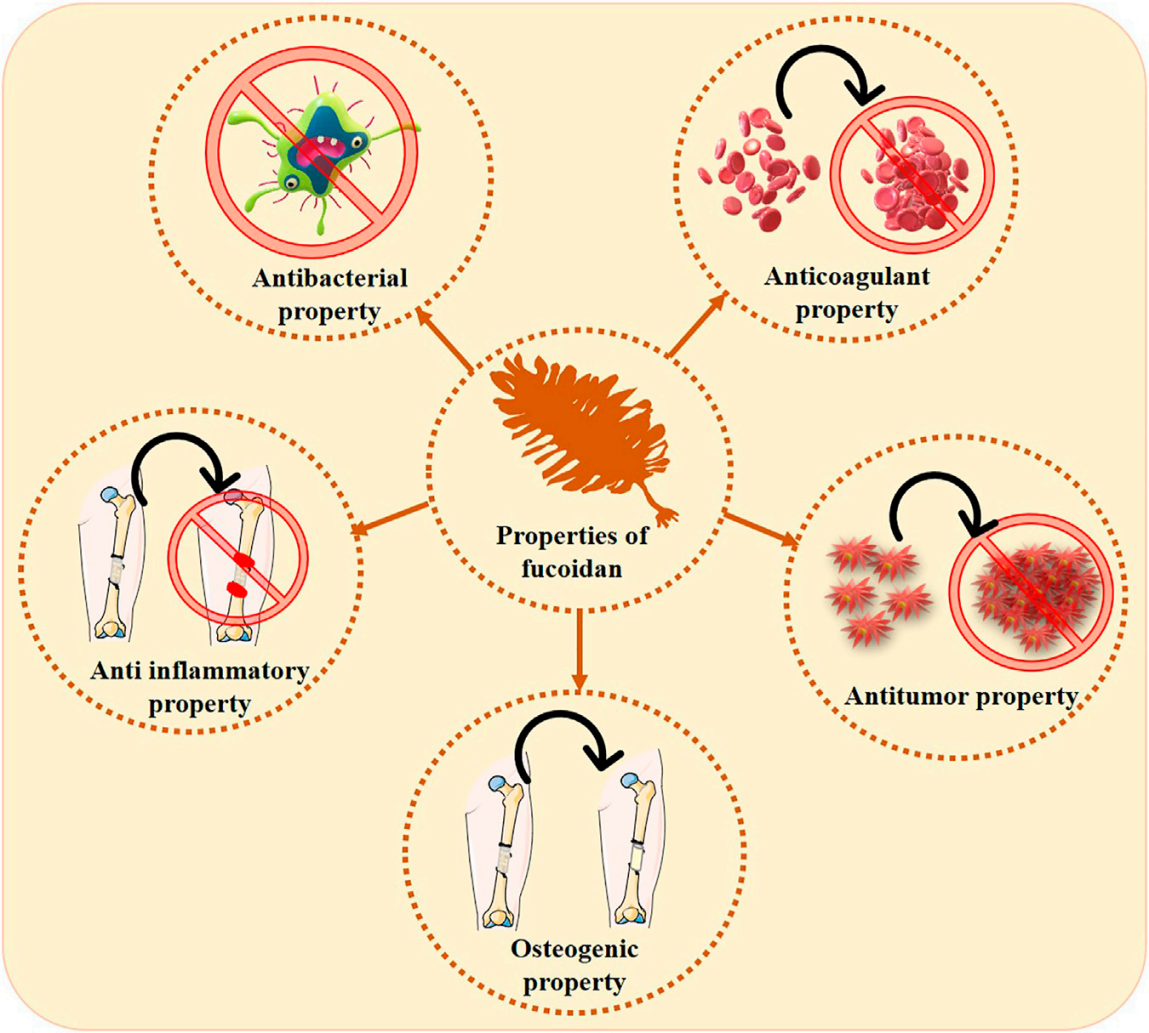
Various properties exhibited by fucoidan pertaining to bone tissue engineering.

Activation of proangiogenic growth factors like FGF is bound and modulated by the seaweed compound fucoidan. Fucoidan improves the characteristics of endothelial cells, reduces the release of plasminogen activator inhibitor-1, and controls cell surface in the presence of growth factor (Joshi et al., 2019). A brief note on various key properties of fucoidan for bone tissue engineering application (Figure 5) has been discussed in the fore coming subsections.

### 3.1 Antibacterial property

Fucoidan has been demonstrated to have good antibacterial properties. Fucoidan has been shown in research by Maria Consuelo and her associates to exhibit antibacterial action against *Listeria* monocytogenes and *Salmonella* enterobacterial activity against *Listeria monocytogenes* and *Salmonella enterica*. The study found that the concentration of fucoidan utilized, the temperature maintained, and the length of time fucoidan was exposed to the pathogens were the key determinants of the bacteriostatic and bactericidal activity against these pathogens (Poveda-Castillo et al., 2018). The antibacterial capability of the *S. aureus* species was identified in a different investigation executed by Ke-Xin Yu’s research team. Using an antibacterial susceptibility test, the study’s findings demonstrated that fucoidan has antimicrobial potential against this species (Genga Raj et al., 2019). Since fucoidan has a lower molecular weight than other antibacterial compounds, it is more effective. This was accomplished by the research team led by Omid Ashayerizadeh, who used depolymerization to create low-molecular-weight fucoidan (Ashayerizadeh et al., 2020). Fucoidan’s antibacterial properties were investigated in human microorganisms like *Vibrio cholera* and *Salmonella typhi* by Thangapandi Marudhupandi and his team. The findings indicate that fucoidan could be utilized as a safe antibiotic for a variety of bacterial illnesses, although further research into the mechanism of action is needed (Marudhupandi and Kumar, 2013). Numerous additional strains, including *E. coli, S. epidermidis, S. aureus*, and *B. licheniformis*, have also been the subject of the study*.* The conclusion was that *E. coli* compared to other bacterial strain, was the most susceptible to fucoidans (Ayrapetyan et al., 2021).

### 3.2 Anti-inflammatory property

The immune system’s primary reaction following an injury is inflammation (Apostolova et al., 2020). Being biocompatible, biodegradable, and having the lowest toxicity possible are all qualities that the optimal scaffold for tissue engineering should have to avoid any inflammatory effects (Venkatesan et al., 2019).

### 3.3 Antitumor and anti-cancerous property

Fucoidan has been discovered to impede angiogenesis and cause cell cycle arrest to inhibit cancer (Zorofchian Moghadamtousi et al., 2014; Jin et al., 2021). According to existing research, fucoidan slows the growth of cancer by interacting with growth factors such as transforming growth factor beta (TGF- β), vascular endothelial growth factor (VEGF), bone morphogenetic protein (BMP), and estrogen receptor and caspase pathways (van Weelden et al., 2019). Even though they have been examined, fucoidan isolated from different species interacts differently and exhibits a variety of unique features. To emphasize these qualities, research is still needed to determine the mechanism of action of fucoidan.

### 3.4 Anticoagulant property

Studies have shown that the anti-inflammatory and anticoagulant properties of fucoidan are interdependent, as well as reliant on the fucose and sulfate concentrations and the backbone chain structure. However, research by Preobrazhenskaya and colleagues demonstrated that these are unrelated and not connected (Ushakova et al., 2009). There are studies where silver nanoparticles were made from fucoidan after it was removed. These nanoparticles demonstrated improved anticoagulant properties, which were investigated using an activated partial thromboplastin time assay. As a result, they can be employed as carriers for the delivery of fucoidan into cells (Shanthi et al., 2021).

### 3.5 Osteogenic property

By shielding them from enzymatic breakdown and improving their presentation to certain receptors, fucoidan is known to boost the actions of several factors, including basic FGF and BMP (Pereira et al., 2011). Fucoidan has also been linked to bone and tissue regeneration in studies. The sulfate ester group of L-fucose, which is present, contributes to the regeneration characteristic (D et al., 2020). This is supported by a recent study that attempted to develop a biomaterial free of xenografts out of poly L-glycolic acid and fucoidan. This study indicated that the scaffolds that included fucoidan had superior osteogenic potential (Yoo et al., 2022). When Igondjo et al. demonstrated that the fucoidan’s presence stimulated the expression of osteogenic differentiation markers, such as alkaline phosphatase, collagen type I, and mineral deposition, and that it also encouraged cell proliferation, they demonstrated the fucoidan’s osteoconductive property. These results are promising with respect to fucoidan as a promising biomaterial to be used for bone regeneration and artificial bone building in clinical use (Igondjo Tchen Changotade et al., 2008).

## 4 Global statuses of fucoidan research in bone

**FIGURE 6:**
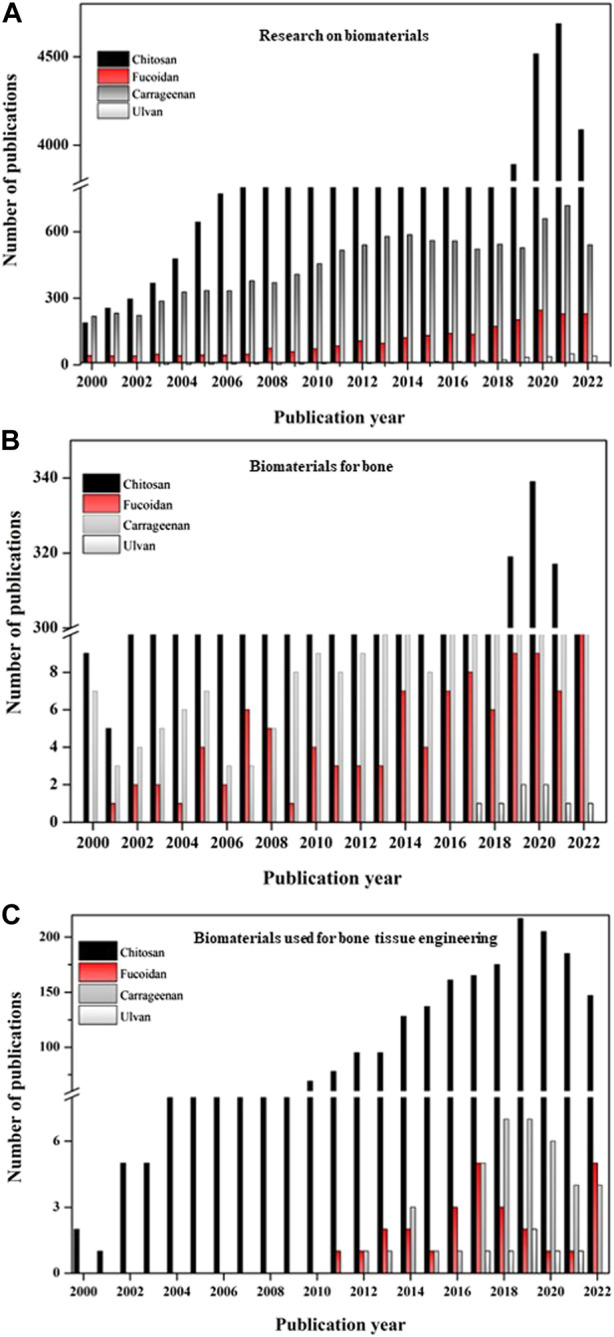
Graph depicting the publication details of the biomaterials from 2000 to 2022. **(A)** Publications on research with various biomaterials (chitosan, fucoidan, carrageenan, ulvan); **(B)** Publications of research with biomaterials (chitosan, fucoidan, carrageenan, ulvan) especially on bone; **(C)** Publications of research with biomaterials (chitosan, fucoidan, carrageenan, ulvan) especially on bone tissue engineering; (Source: PUBMED; keywords chosen: chitosan, fucoidan, carrageenan, ulvan; chitosan for bone, fucoidan for bone, carrageenan for bone, ulvan for bone, chitosan for bone tissue engineering, fucoidan for bone tissue engineering, carrageenan for bone tissue engineering, ulvan for bone tissue engineering; Accessed: 14 15 November 2022).

The database used to learn about the status of fucoidan for bone research globally is PUBMED. It has also been compared to research on ulvan and carrageenan, two additional sulfated polysaccharides. The global research on fucoidan has also been compared to the often-researched polysaccharide chitosan in order to better understand the need for study and the research opportunities accessible with this polysaccharide for bone. Data from a global study of the biomaterial’s chitosan, fucoidan, carrageenan, and ulvan are shown in Figure 6. The sulfated polysaccharide’s research findings have been contrasted with those of the highly studied polysaccharide chitosan in order to learn more about them.

The complete investigation with the biomaterials—chitosan, fucoidan, carrageenan, and ulvan—is shown in Figure 6A. Although it can be assumed that research on fucoidan has existed since the 2000’s, the study focusing on bone tissue engineering only began in 2010. Figure 6B suggests that research on these biomaterials for bones has advanced over years and has suddenly expanded during the past 5 years. Fucoidan’s interest in bone tissue engineering applications has grown significantly during the past 10 years, matching that of another sulfated polysaccharide, carrageenan (Figure 6C).

## 5 Fucoidan for bone tissue engineering

**FIGURE 7:**
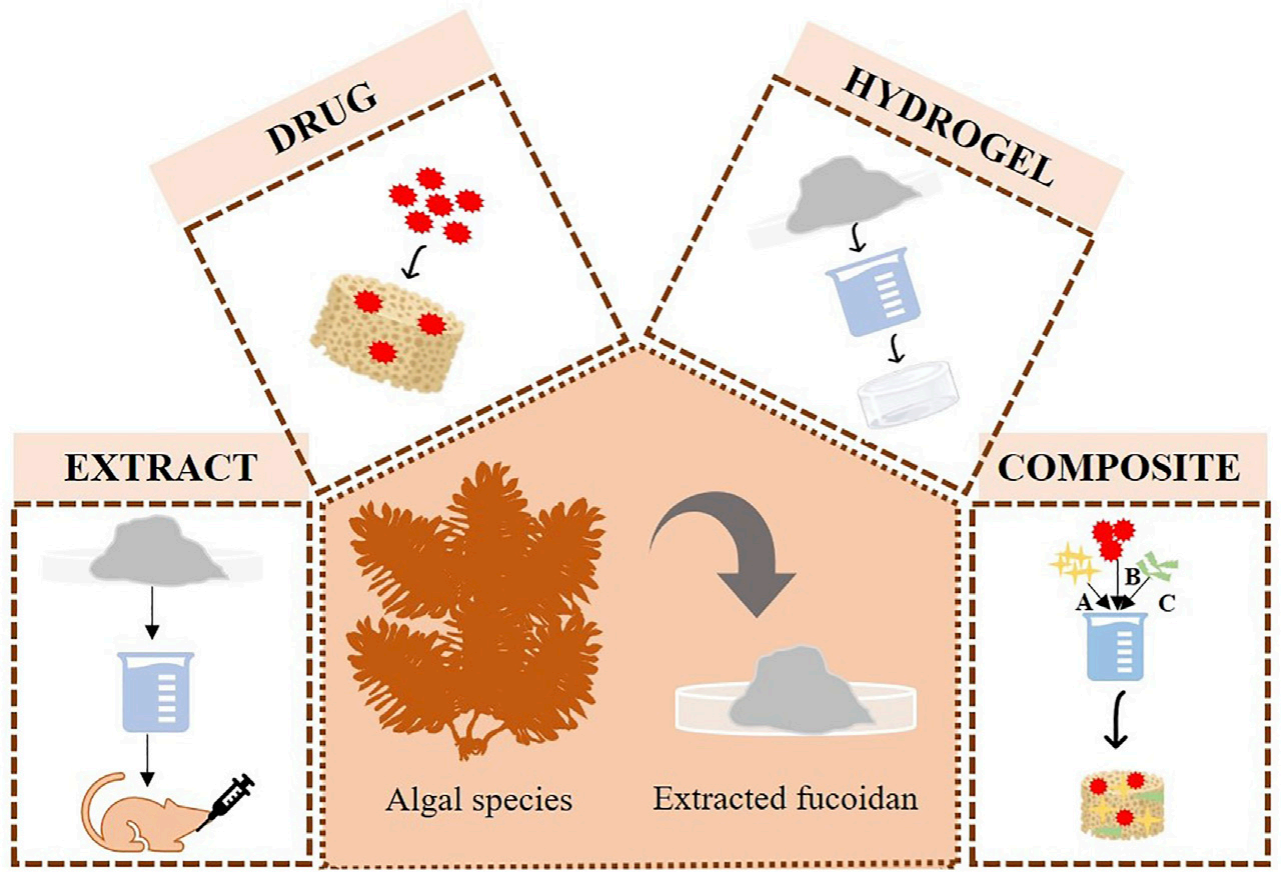
Various forms in which fucoidan is used in bone tissue engineering application.

This biomaterial, fucoidan, has been shown to promote the expression of type 1 collagen, osteocalcin, and BMP2 and aid in mineral deposition related to bone mineralization (Eshwar et al., 2021). Various forms in which fucoidan could be used for bone tissue engineering have been discussed in the upcoming session (Figure 7). As depicted in the figure, this biomaterial can be used as an extract, as a drug combined with other biomaterials to release it when needed to demonstrate its properties, as a component of hydrogels that can hold a variety of properties, including injectability and swelling ability, and as a final option, as a component of composite scaffolds that can offer sufficient mechanical properties while still giving the properties necessary for bone regeneration. These are covered in further detail in the ensuing subsections.

### 5.1 Fucoidan as drug

**FIGURE 8:**
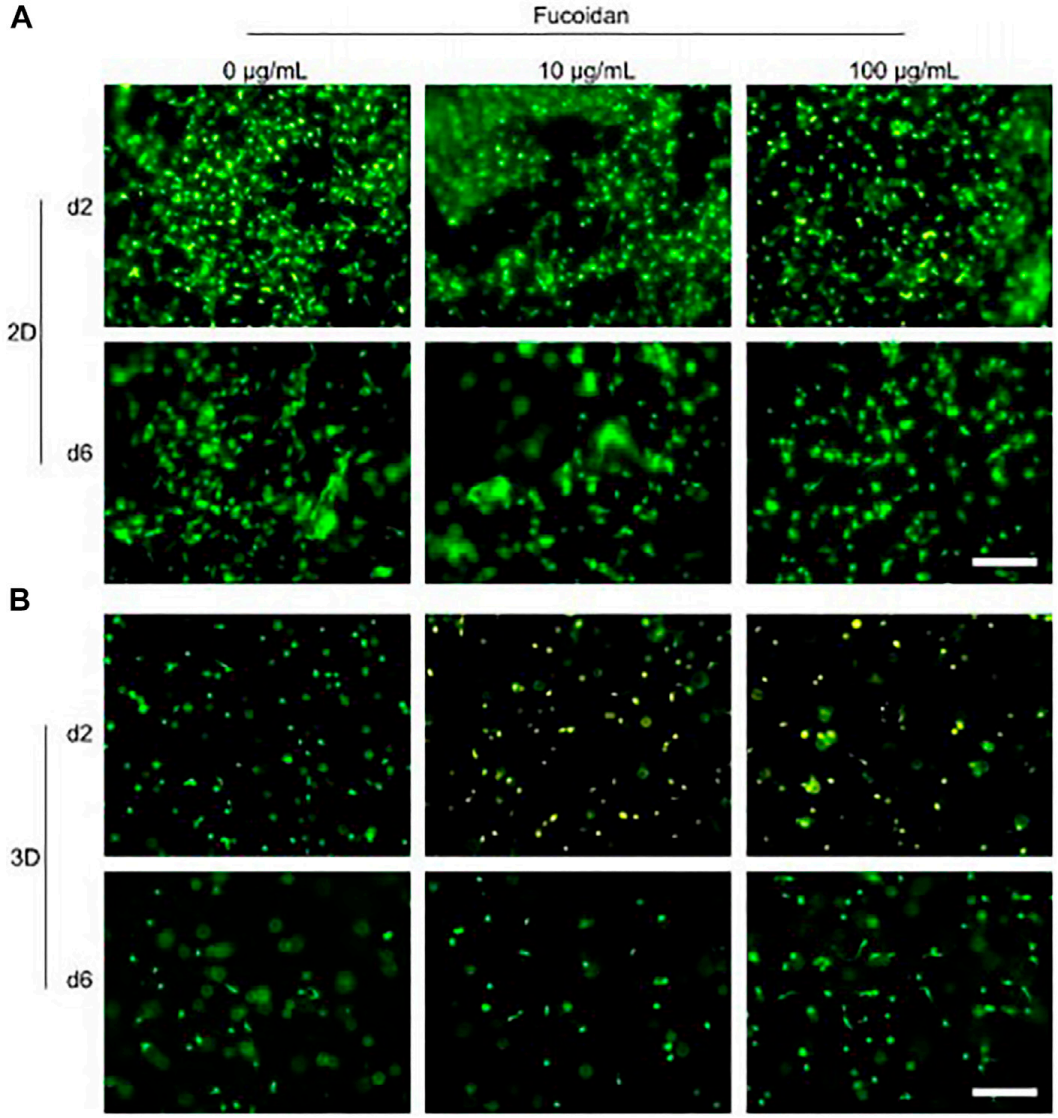
Injectable chitosan collagen hydrogel containing fucoidan at varying concentrations checked for its biocompatibility. **(A)** and **(B)** shows the biocompatibility of the hydrogel when cells were cultured on top of hydrogel (2D culture and when they were encapsulated with the hydrogel as 3D culture respectively. [Reproduced with permission from Ref (Ohmes et al., 2022b) under Creative Commons Attribution License (CC BY)].

Fucoidan was given in a study by Ohmes et al. (2022b) using a thermoresponsive injectable hydrogel comprised of chitosan, collagen, and beta-glycerophosphate. It has thus been regarded in this case as a drug, and its release profile revealed that 60% and 80% of it were released after the time points of 2 and 6 days, respectively. Mesenchymal stem cells (MSCs) were also used in the assays, and it was discovered that they were biocompatible (Figure 8) and could aid in cell proliferation This study has demonstrated the potential use of fucoidan as a medication that can be delivered through several types of carriers, such as hydrogel, for bone tissue engineering applications.

**FIGURE 9:**
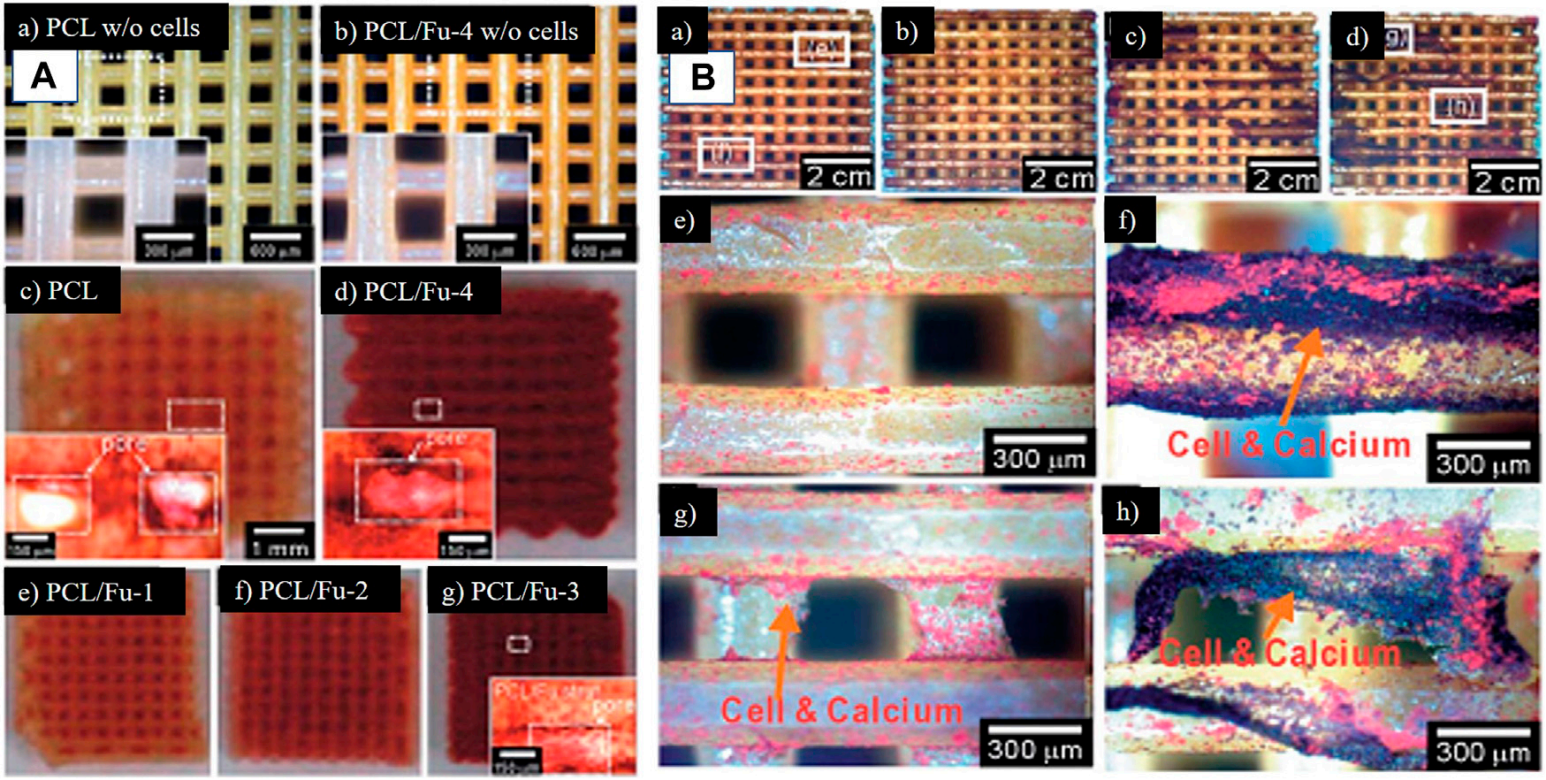
Optical images of 3D printed scaffolds. **(A)** (a) PCL (b) PCL + fucoidan 20% scaffolds after being incubated in the medium for 7 days without cells. THe yellow color is fucoidan which is evident in (b) (c–g) are the images of ARS staining for these scaffolds scaffolds after 14 days of cell culture. **(B)** ARS staining to confirm mineralization (a) PCL-alginate scaffold, (b) PCL + alginate 1% + fucoidan 2%, (c) PCL + alginate 3% + fucoidan 2%, and (d) PCL + alginate 5% + fucoidan 2% at day 14. (e–h) are magnified images of (a) and (d), respectively. (Reproduced with permission from Ref (Jin and Kim, 2011; Jin and Kim, 2012). Copyright 1991, 2005, Royal Society of Chemistry).

Recent times have seen greater technological advancements, with rapid prototyping being one of them. A composite scaffold comprised of polycaprolactone and fucoidan was created using this method. Fucoidan’s impact on osteoblast-like cell proliferation and mineral deposition was also studied utilizing a range of fucoidan concentrations. Finally, the augmentation of cellular proliferation and mineral deposition has been seen in conjunction with the first burst release of fucoidan and the subsequent continuous release (Jin and Kim, 2011). The same group attempted to control the release profile of fucoidan by first constructing polycaprolactone structures and then covering it with a mixture of alginate and fucoidan because burst release was detected in the study mentioned above. The results of this approach demonstrated active biological capabilities for promoting bone regeneration in comparison to the polycaprolactone and fucoidan composite without release or with burst release of fucoidan (Jin and Kim, 2012). The biomineralization studies of the scaffold made of polycaprolactone and fucoidan and polycaprolactone with a mixture of alginate and fucoidan have been shown in Figure 9.

### 5.2 Fucoidan as extract

The study team led by Se-Kwon Kim first used fucoidan as an extract to assess its osteogenic potential back in 2009. Fucoidan was tested for its effects in this research on MG-63 cell lines, which were discovered to be an appropriate candidate for osteogenesis by the outcomes of numerous assays to determine its osteogenic potential as well as through the phenotypic indicators of early stage and terminal differentiation (Cho et al., 2009).

**FIGURE 10:**
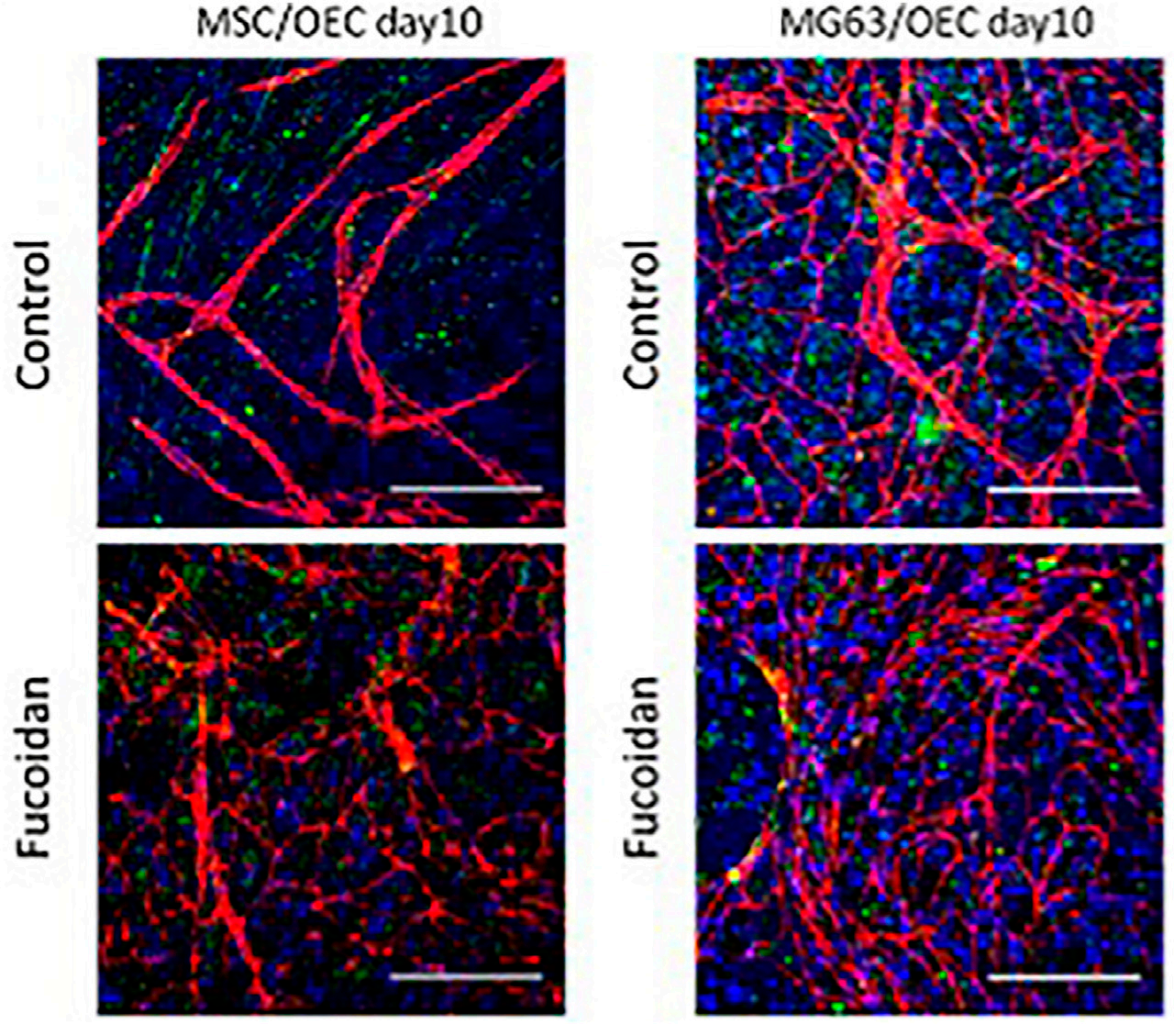
The anti-angiogenic potential of fucoidan. [Reproduced with permission from Ref (Ohmes et al., 2022b) under Creative Commons Attribution License (CC BY)].

The research team of Sabine Fuchs has also tested the bioactivity of fucoidan as an extract. In the most recent study, fucoidanase hydrolysis was used as an extraction technique, enhancing the bioactivity of the isolated fucoidan. Additionally, they have removed the low and medium molecular weight fucoidans that, in contrast to high molecular weight fucoidans, did not inhibit bone stem cells’ synthesis of pro-angiogenic molecules (Ohmes et al., 2022a). The same group also discussed the outcomes of enzymatic fucoidan extraction and how its high concentration of fucose and sulfate content could adversely affect the angiogenic and osteogenic processes in isolated human outgrowth endothelial cells and differentiated osteoblast-like lineage mesenchymal stem cells (Ohmes et al., 2020). The same group also considered how to make use of this unfavorable outcome and arrived at the opinion that aggressive angiogenesis in osteosarcoma should be limited. This suggests that the metabolic activity of endothelial cells and osteoblast cells is concentration-dependent. To determine the dose of fucoidan needed to prevent vascularization, the tests were first carried out in monocultures of human blood-derived outgrowth endothelial cells (OECs), mesenchymal cells, and MG-63 cell lines. Cocultures of MG-63 cells and mesenchymal cells with human blood-derived outgrowth endothelial cells were then carried out to ascertain the effect of fucoidan on reducing vascularization in bone tumors like osteosarcoma (Wang et al., 2017). This property of fucoidan has been studied in the coculture of endothelial cells and osteoblast cells as shown in Figure 10. From the confocal image, it could be inferred that the control with cocultured cells showed the formation of angiogenic structures whereas the one treated with fucoidan has only a few angiogenic structures thereby confirming that the presence of fucoidan could inhibit the abrupt formation of angiogenic structures in cases of cancer. In light of this, it could be thus argued that fucoidan can be employed as a scaffolding material to replace bone that has been lost due to osteosarcoma, thereby halting the growth of angiogenesis.

The sulfated polysaccharide, low-molecular-weight fucoidan enhances the expression of several growth factors, including pro-angiogenic growth factors. Jessica Pereira and colleagues pre-treated human adipose tissue-derived stem cells with low-molecular-weight fucoidan and compared this combination to low-molecular-weight heparin preconditioned human adipose tissue-derived stem cells. The findings of this comparative investigation demonstrated that low-molecular-weight fucoidan treatment had an advantageous effect on osteogenic differentiation rather than phenotypic changes in the cells. However, in heparin-treated cells, no bone growth was visible after 5 or 8 weeks, perhaps as a result of cell death, putting an end to the potential of fucoidan for enhancing bone regeneration (Pereira et al., 2011).

These low-molecular-weight fucoidans, as we previously described, induce angiogenesis both *in vitro* and *in vivo*. They also enhance collagen production and fibroblast growth. These characteristics are mostly attributable to these fucoidans’ capacity to protect and promote the signaling of heparin-binding growth factors, including FGFs and VEGF. By pre-treating the commercial scaffold with fucoidan and then conducting the assays to detect bone regeneration, the osteoconductive property of fucoidan has been examined. The results of this research demonstrate the value of this biomaterial for bone (Igondjo Tchen Changotade et al., 2008).

Numerous other features, in addition to the osteogenic, osteoconductive, and angiogenic ones, also play a significant role. Among these are the shape of the cells and their movement. Fucoidan’s effect on these properties has been studied for the cells in the presence of this property by Kim et al. (2015). It also has the ability to prevent osteoclast differentiation. This has been studied by Jin et al. (2017) and the findings indicate that fucoidan’s ability to treat osteoporosis can be used. Preclinical testing of this has also been done on rats with ovariectomies.

### 5.3 Fucoidan as a component of nanocomposite scaffolds

Venkatesan and colleagues developed a composite scaffold out of chitosan, alginate, and fucoidan, utilizing freeze-drying as a replacement for the bone graft. As a result of a significant improvement in cytocompatibility, cell proliferation, and alkaline phosphatase secretion, as well as a twofold increase in protein adsorption and mineralization in the scaffolds with fucoidan as a component compared to the other control scaffold made of chitosan and alginate, *in vitro* studies revealed the osteogenic potential (Venkatesan et al., 2014). The same group also concluded that hydroxyapatite and fucoidan together make a viable scaffold for bone tissue construction (Jeong et al., 2013). Ipsita Banerjee and her team of researchers constructed a new composite scaffold out of fucoidan, gelatin, and tricalcium phosphate after learning that fucoidan can make superior scaffolds with alginate, tricalcium phosphate, and other polymers. Peptide chains were also added to the scaffold. This scaffold, as anticipated, demonstrated a considerable increase in osteogenic potential, making it an excellent choice for bone tissue engineering (Pajovich and Banerjee, 2017).

The composite scaffold comprised of chitosan, hydroxyapatite, and fucoidan has been discussed by Lowe et al. (2016). The outcomes of this composite were much better than those of the chitosan, chitosan and fucoidan, and chitosan and hydroxyapatite control scaffolds. Mesenchymal stem cells generated from periosteum were used in *in vitro* tests, and the findings showed that the scaffolds containing fucoidan had superior biocompatibility and biomineralization than the other control scaffolds. Fucoidan was found to increase biomineralization, which led researchers to believe that it would be a promising biomaterial for bone tissue regeneration. A composite scaffold was developed by the research team of Hanumantharao by freeze-drying chitosan, tricalcium phosphate, and fucoidan. The results for these scaffolds indicated an increase in osteocalcin levels, a major marker, indicating their potential (Puvaneswary et al., 2015). The same research has also found that the presence of fucoidan prompted human mesenchymal stem cells to differentiate into osteogenic-like cells, which contributed to the scaffold’s osteogenic nature (Puvaneswary et al., 2016).

The modified chitosan known as N, O-carboxymethyl chitosan has also been conjugated with fucoidan to test its biomineralization capabilities. This conjugated scaffold produced results similar to those achieved with the inclusion of chitosan, demonstrating its potential (Lu et al., 2018). The same team continued to experiment with different combinations of ingredients, such as nano-hydroxyapatite, hydroxypropyl chitosan, and fucoidan, that had been adsorbed in them. This study also demonstrated that the fucoidan-adsorbed scaffolds had improved 7F2 osteoblast cell performance (Lu et al., 2019).

### 5.4 Fucoidan as a component of the hydrogel

The use of hydrogels in bone tissue engineering has always been intriguing. Composite hydrogel studies have been and continue to be conducted in order to increase osteogenic potential. One of the components of the hydrogel for this application also includes fucoidan. Shruthi Eshwar’s research team recently investigated and evaluated an injectable hydrogel consisting of fucoidan, gelatin, and chitosan. Preclinical research revealed that the animal group treated with fucoidan-containing hydrogel had better new bone formation per total area than the control group. These tests were run on Wistar rats and observed for 28 days, demonstrating their effectiveness (Eshwar et al., 2021).

Usually, modifications are made to the biomaterial to enhance its properties. In one of the articles by Pai-An Hwang and the group, fucoidan has been methacrylated and grafted with methacrylated hyaluronan to create a photocrosslinkable hydrogel. This hydrogel was examined for its capacity to promote mineralization and cell adhesion in MG-63 cells, hence demonstrating the potential of fucoidan (Hsu et al., 2021).

## 6 Preclinical studies of fucoidan for bone

Preclinical research serves as a bridge between benchwork and clinical research, advancing the product’s use at the bedside. For these studies to be successful, a large amount of data is required, and it is collected to show the stability, effectiveness, compatibility, and safety of animals and humans throughout the study (Shegokar, 2020). *In vitro*, *in vivo*, *ex vivo*, or *in silico* can all be used to do this; the choice is dependent upon the study that will be carried out. The preclinical study on fucoidan which was especially used for bone tissue creation is covered in great length in this section.

### 6.1 *In vitro* studies

“*In vitro*” is a Latin term that means “within the glass” and refers to research done on cell cultures outside of the human body. *In vitro* research has various advantages. The main advantage of this is that no animals or people are involved, minimizing the harm to living things. They are also considerably less expensive and produce trustworthy and effective outcomes. Additionally, it is possible to mimic the microenvironment of human tissues (Moore, 2021). Following is a brief discussion of a few studies where fucoidan has been preclinically investigated for use in bone-related applications.

A nanocomposite scaffold made of chitosan, nanohydroxyapatite, and fucoidan was created by Lowe et al. (2016) and further examined by mesenchymal stem cells generated from the periosteum. The findings showed that these cells have excellent biomineralization on the scaffold and are biocompatible. A group of scientists led by Kwack et al. (2015) fabricated fucoidan and polydopamine composite film to evaluate if the synthetic composite can provide microenvironments to differentiate the periodontal ligament stem cells. Tae Young et al. (2016) researched to understand the specificity of how stem cells interact with a composite made of hydroxyapatite and fucoidan. Another recent study by Devi G.V. et al. (2022a) examined the capacity of mesenchymal stem cells to differentiate into the bone using a composite scaffold that contains fucoidan. Studies on its impact on osteoblastic MG-63 differentiation came to the conclusion that it helped to increase the activity of alkaline phosphatase and osteocalcin, which are, respectively, the phenotypic markers for early-stage and terminal osteoblastic differentiation (Cho et al., 2009). To test the potential of nano-hydroxyapatite with hydroxypropyl chitosan composite scaffolds with fucoidan adsorbed on them, *in vitro* tests with 7F2 osteoblast cells were also conducted. Since the results of *in vitro* tests revealed higher calcium deposition and alkaline phosphatase activity, confirming mineralization, this composite was determined to have the potential for use in bone tissue engineering (Lu et al., 2019). A composite made of alginate, hydroxyapatite, graphene oxide, and fucoidan may be used in place of bone grafts in orthopedic applications due to its favorable physical, structural, and osteogenic potential. In addition to assisting in osteogenesis, the ingredient fucoidan also helps to suppress osteoclast cells. This was supported by a study by Jin and colleagues, which discovered that fucoidan inhibited the Receptor activator of nuclear factor kappa-Β ligand (RANKL) pathway, which in turn prevented osteoclast activity and decreased bone resorption (Jin et al., 2017). As a result, it sheds insight into a possible application for the treatment of osteoporosis. Although many studies concentrated on the osteoblastic activity that demonstrates its mineralization, Kim et al. (2015)’s research was concentrated on cell morphology and migration. They demonstrated that fucoidan-treated cells migrated more than control cells did, but there was only a slight change in the morphology of these cells.

### 6.2 *In vivo* studies

The Latin word “*in vivo*” means studies performed within living organisms. The main disadvantage of *in vitro* studies is the inability to examine the effect on the entire body rather than just cells (Moore, 2021). *In vivo* models have been used to overcome this. However, they suffer from ethical problems, which continue to be a top concern in research. Although an extensive study has been done on fucoidan recently, relatively little has been done up until the *in vivo* level to gauge its effectiveness for bone tissue engineering.

In an attempt to assess the osteogenic capacity of human adipose tissue-derived stem cells loaded with dicalcium phosphates and low molecular weight fucoidan, Dominique Helley and colleagues (Pereira et al., 2011), the *in vivo* study for this utilized a mouse model of ectopic bone development to assess the osteoinductivity of stem cells generated from human adipose tissue in conjunction with dicalcium phosphate. Even though the study focused on osteogenic differentiation, it was discovered that fucoidan aids in angiogenesis, and more research is needed to determine the mechanism underlying this ability. Nielsen et al. (2022)’s research team recently examined the effectiveness of extracted fucoidan in bone regeneration and bone fixation, contrasting test groups using hydroxyapatite and fucoidan with allografts in a sheep model. After imaging, histomorphometry, and mechanical investigation, it was determined that hydroxyapatite and fucoidan together produced outcomes similar to those of allografts. Through the use of a rabbit bone model, the Tae Young et al. (2016) has further supported their *in vitro* inquiry into the effects of fucoidan on biomineralization.

From all the studies stated above, it is clear that fucoidan has been essential in promoting osteogenesis and angiogenesis. Due to the lack of research on composites for bone tissue engineering, further research is needed before this biomaterial may be further commercialized.

A summary of the preclinical study carried out on fucoidan for bone has been shown in Table 1.

**TABLE 1:** Preclinical study carried out on fucoidan for bone.

S No	Preclinical study	Fucoidan administration	Animal used/cells used	Study period	Aim of study	References
1	*In vivo*	Extract of fucoidan	Sprague-Dawley (SD) rats	8 weeks	Inhibit osteoclast precursor differentiation, maturation and bone resorption	Jin et al. (2017)
2	*In vitro*	Extract of fucoidan	MG-63 cell lines	—	Osteoblastic cell differentiation	Cho et al. (2009)
3	*In vitro*	Extract of fucoidan	Mesenchymal stem cells	21 days	Osteogenic differentiation potential in mesenchymal stem cells	Devi G.V. et al., (2022a)
4	*In vitro*	Fucoidan incorporated composite scaffold	Mesenchymal stem cells	21 days	Osteogenic differentiation of mesenchymal stem cells	Devi G.V. et al., (2022b)
5	*In vitro*	Fucoidan/polydopamine composite modified surface	Periodontal ligament stem cells	7 days	Osteogenic potential	Kwack et al. (2015)
6	*In vitro*	Chitosan-natural nano hydroxyapatite-fucoidan nanocomposites	Periosteum-derived mesenchymal stem cells	28 days	Bone tissue engineering	Lowe et al. (2016)
7	*In vitro*/*In vivo*	Nano hydroxyapatite-fucoidan bio nanocomposites	Mesenchymal stem cells/Rabbit’s tibia model	8 weeks	Bone regeneration	Tae Young et al. (2016)
8	*In vitro*/*In vivo*	Low molecular weight fucoidan	Human adipose derived stromal cells | Mouse model	7 days/8 weeks	Osteogenic capacity	Pereira et al. (2011)
9	*In vivo*	Hydroxyapatite, fucoidan	Sheep femurs	12 weeks	Bone regeneration	Nielsen et al. (2022)

## 7 Other applications of fucoidan

Even though the article is primarily focused on the usage of bone tissue engineering, fucoidan has also been investigated for several other applications, including cancer, wound healing, cardiovascular and vascular tissue engineering, and immunomodulation.

The ability of fucoidan to promote angiogenesis. The investigation of neovascularization has made use of this characteristic. For instance, the study of Agung Purnama et al., was based on it, showing that fucoidan interacts with VEGF in 3D scaffolds to promote neovascularization. Mice were also used in the investigation. The findings showed that subcutaneous implantation of fucoidan-loaded hydrogel in mice caused the establishment of a significant vascular network with increased vessel density when compared to unmodified hydrogels (Purnama et al., 2015).

Vascular tissue engineering has also been a focus, similar to the use of fucoidan in bone tissue creation. Fucoidan, chitosan, and poly(vinyl alcohol) scaffolds were utilized in a work by Zhang et al. (2017), to replicate the ECM and match the mechanical properties of blood vessels. The material’s physicochemical, mechanical, and biological characteristics suggested that it may be used for vascular tissue engineering.

The area of cancer has also been extensively researched with fucoidan as a biomaterial. Fucoidan-coated gold nanorods were coupled with anti-epidermal growth factor receptor monoclonal antibodies as a novel photothermal ablation agent for cancer nanotheranostics in research by Panchanathan Manivasagan et al. In this way, the agent served as a prospective agent for use in cancer nanotheranostics in the future (Manivasagan et al., 2017). It has also been synthesized as nanoparticles along with chitosan, which was then used to transport the anti-cancerous model drug gemcitabine. When employed as a delivery method against human breast cancer cells, this nanoparticle technology specifically targeted the malignant cells while having no adverse effects on endothelium cells.

One of the common chronic illnesses, that is widespread around the world is diabetes. As a treatment method Lara L. Reys and team have investigated with the use of methacrylated fucoidan, a modified kind of fucoidan, was employed to create fucoidan hydrogel particles. These particles were then incubated with human pancreatic cells, and the results showed that they could help with the development of pseudo-islets and 80% of the viability of the cells. This finding opens the door for its possible usage as an insulin delivery vehicle (Reys et al., 2022). Therefore, we could conclude that fucoidan’s use as a biomaterial has never been restricted for bone tissue engineering, but that due to its wide range of qualities, it has been investigated for a wide range of other uses, such as the few that were mentioned above.

## 8 Conclusion and future perspectives

Although the polysaccharide fucoidan was discovered and utilized in medicine in the 1950’s, its usage in bone tissue engineering has been fairly restricted. As was said in the worldwide status of research, there has been scant research with this polysaccharide on bone tissue engineering, but it has been researched to some extent for cancer. Since the polysaccharide’s overall potential is known at this point, an intensive study that can open new doors for a variety of applications is desperately needed. This polysaccharide, which contains several essential properties, could be investigated for bone tissue engineering in order to create a scaffold that has the potential to increase osteogenic activity. It also has additional benefits like anti-inflammatory and anti-bacterial properties that help combat the major drawback of infections. This is one of the biomaterials that has not been extensively studied and has not yet entered the clinical trial stage, providing new research opportunities for up-and-coming scientists.

Recent studies on the rheological properties of composites containing fucoidan have been sparse due to the rapid advancement of scaffold fabrication technology (Carvalho et al., 2021). This paves the way for more investigation into the creation of bio inks for 3D bioprinting. Its high-water solubility is one of the main difficulties in employing fucoidan. A key objective of the research is to slow down the degradation of fucoidan, which can be done by changing the hydrophilic chains in them or moving toward conjugation with other composites. Hence, by overcoming such known drawbacks this biomaterial can be explored more to get more benefits out of it.

## Abbreviations

IUPAC, International Union of Pure and Applied Chemistry; FGF, fibroblast growth factor; BMP, Bone morphogenetic protein; VEGF, vascular endothelial growth factor; TGF-β, transforming growth factor beta; RANKL, Receptor activator of nuclear factor kappa-Β ligand; MSCs, Mesenchymal stem cells; OECs, human blood derived outgrowth endothelial cells; kDa, kilodaltons; 3D, 3 Dimension.
